# Development of Eighteen Microsatellite Markers in *Anemone amurensis* (Ranunculaceae) and Cross-Amplification in Congeneric Species

**DOI:** 10.3390/ijms13044889

**Published:** 2012-04-18

**Authors:** Mingzhou Sun, Xiao Yin, Fengxue Shi, Lin Li, Mingrui Li, Linfeng Li, Hongxing Xiao

**Affiliations:** Key Laboratory of Molecular Epigenetics of Ministry of Education, Northeast Normal University, Changchun 130024, China; E-Mails: sunmingzhou@hotmail.com (M.S.); yinx458@nenu.edu.cn (X.Y.); shifx816@nenu.edu.cn (F.S.); lil400@nenu.edu.cn (Lin.L.); limr145@nenu.edu.cn (M.L.); lilf241@nenu.edu.cn (Linf.L.)

**Keywords:** *Anemone*, *A. amurensis*, microsatellite, polyploidy, polymorphism information content

## Abstract

Polyploidy plays an important role in the evolution of plant genomes. To enable the investigation of the polyploidy events within the genus *Anemone*, we developed eighteen microsatellite markers from the hexaploid species *A. amurensis* (Ranunculaceae), and tested their transferability in five closely related species. The number of total alleles (N_A_) for each resulting locus varied from one to eight. The polymorphism information content (PIC) and Nei’s genetic diversity (N_GD_) for these microsatellites ranged from 0.00 to 0.71 and 0.00 to 0.91, respectively. For each population, the N_A_ was one to seven, and the values of PIC and N_GD_ varied from 0.00 to 0.84 and 0.00 to 0.95, respectively. In addition, most of these microsatellites can be amplified successfully in the congeneric species. These microsatellite primers provide us an opportunity to study the polyploid evolution in the genus *Anemone*.

## 1. Introduction

Polyploidy has been recognized as a pervasive force in plant evolution, and more than half of flowering plants ultimately have a polyploid ancestry [1,2]. Those polyploidy events therefore contributed greatly to the diversification and evolution of angiosperms [3,4]. In this study, in order to evaluate the polyploidy events within the genus *Anemone*, we investigated an annual herbaceous plant of northeastern China, namely *Anemone amurensis* Kom. (*Ranunculaceae*). According to previous studies, the species within the genus *Anemone* not only show high morphological diversity, but also occupy a wide range of habitats, including alpine tundra, woodlands and semidesert [5,6]. It is demonstrated that the species within the genus *Anemone* has been reported to encompass diploid, tetraploid and hexaploid chromosomal races [5,7,8]. For example, although the species *A. amurensis* has a hexaploid genome, its closely related species were found to have diploid and tetraploid genomes. These attributes indicate that the genus *Anemone* is an ideal system to study polyploidy. Nonetheless, studies of polyploidy in this genus are still limited due to the lack of suitable and efficient molecular markers. Microsatellite markers are appropriate candidates for studying this non-model species as they are highly polymorphic and co-dominant. We therefore developed eighteen microsatellite markers from *A. amurensis* and tested their transferability in other congeneric species. These microsatellites provide us with an opportunity to study polyploidy events within the genus *Anemone*.

## 2. Results and Discussion

In total, 106 positive clones were sequenced on an ABI 3730 DNA sequencer (Applied Biosystems, Foster City, CA, USA), of which 74 clones were found to have microsatellite motifs. Thirty-three of these clones were chosen to design primer pairs, and 18 of these produced single clean amplicons of the expected size (Table 1). The Genbank accession numbers, primer sequences, repeat motifs, size of cloned allele, annealing temperature, number of alleles (N_A_), Nei’s genetic diversity (N_GD_) and polymorphism information content (PIC) can be found listed in Table 1.

Polymorphism and transferability of the 18 microsatellite loci were assessed in 52 individuals of *A. amurensis* and five other closely related species. According to the results, 11 of these microsatellites exhibited polymorphic patterns in the species *A. amurensis*. N_A_ for each locus was between one and eight, and the values of PIC and N_GD_ for each locus varied from 0.00 to 0.71 and 0.00 to 0.91, respectively (Table 1). In addition, the N_A_ for each population ranged from one to seven, and the values of PIC and N_GD_ for each population varied from 0.00 to 0.84 and 0.00 to 0.95, respectively (Table 2). Only the locus HS199 showed a significant departure (*p* < 0.01) from HWE under the diploid model and no significantly (*p* < 0.01) linkage disequilibrium was detected for any pair of loci. For cross-amplification, eight, nine, 13, 14 and 17 of these microsatellites successfully amplified the target regions in four individuals of each of the species *A. silvestris*, *A. raddeana*, *A. cathayensis*, *A. umbrosa* and *A. reflexa*, respectively (Table 3). The N_A_ of these markers in the five congeneric species ranged from one to five.

## 3. Experimental Section

### 3.1. Isolation of Microsatellite Markers

Genomic DNA was extracted from dried leaves of one individual of *A. amurensis* (voucher specimen: NENU20110420001) using the Plant Genomic DNA kit (TianGen, Beijing, China) following the manufacturer’s protocols. Genomic libraries enriched for microsatellite motifs were constructed as described in detail in Zane *et al.* [9]. Briefly, about 300 ng of genomic DNA was digested with the restriction enzyme *Mse* I (New England Biolabs, Beverly, MA, USA) and ligated to double-stranded linkers (5′-TACCAGGACTCAT-3′/5′-GACGATGAGTCCTGAG-3′) with T4 ligase (Fermentas, Burlington, Ontario, Canada) [10]. Then, the diluted disgestion-ligation mixture (1:10) was amplified using MseI-N (5′-GATGAGTCCTGAGTAAN-3′) as primer. The following polymerase chain reaction (PCR) temperature profile was used: 5 min at 94 °C, followed by 20 cycles of 94 °C for 30 s, 53 °C for 1 min, 72 °C for 1 min, and a final extension of 72 °C for 5 min. The reaction mixture contained 1.2 ng diluted disgestion-ligation DNA, 1 × PCR buffer, 2.5 mM Mg^2+^, 0.2 mM of each dNTP, 1 μM of the MseI-N primer and 1 U of Taq polymerase (Takara, Liaoning, China).

For enrichment, the PCR products were denatured and hybridized to the 5′-biotinylated oligo probe (AC)_15_ and these DNA molecules containing microsatellite motifs were captured by streptavidin-coated magnetic beads (Promega, Madison, WI, USA). These recovered DNA fragments were amplified with MseI-N primer and the composition of the PCR reaction mixture as well as the PCR cycling conditions were the same as described above. These PCR products were then ligated into the pMD-18 vector (Takara) and transformed into *Escherichia coli* DH5α competent cells (Takara). The positive clones were picked out and tested by PCR using (AC)_10_ and M13+/M13− as primers, respectively. The PCRs were set up in total volumes of 15 μL, containing 2 μL template DNA, 1 × PCR buffer, 2.5 mM Mg^2+^, 0.2 mM of each dNTP, 0.5 μM of each primer and 1 U of Taq polymerase (Takara). The following PCR cycling profile was used: initial denaturation at 94 °C for 5 min; 30 cycles of 94 °C for 30 s, 55 °C for 30 s, 72 °C for 30 s; and a final extension at 72 °C for 8 min. These clones with sufficient flanking regions (at least 30 base pair in length) were chosen to design primer pairs using the Primer Premier software (http://www.premierbiosoft.com). The conditions of primer designing were performed according to Li *et al.* [11], and each forward primer was fluorescent labeled with either FAM or HEX (Invitrogen, Beijing, China).

### 3.2. Detection of Polymorphism and Data Analysis

Polymorphism was tested in 52 individuals from three populations of *A. amurensis* (Langxiang, 47.062° N, 128.886° E, voucher specimen: NENU20110430001; Dunhua, 43.809° N, 128.120° E, voucher specimen: NENU20110420002; Kuandian, 41.337° N, 124.825° E, voucher specimen: NENU20110426019), and the transferability of these markers were assessed in four individuals of each *A. raddeana* Regel. (voucher specimen: NENU20110426011), *A. silvestris* L. (voucher specimen: NENU20110525009), *A. umbrosa* Mey (voucher specimen: NENU20110512003), *A. reflexa* Stephan (voucher specimen: NENU20110512008), and *A. cathayensis* Kitag (voucher specimen: NENU20110422001) (Table 2). Amplification reactions were carried out in 20 μL reaction volume containing 50 ng template DNA, 1 × PCR buffer, 2.5 mM Mg^2+^, 0.2 mM of each dNTP, 0.5 μM of each forward and reverse primer, and 1 unit of Taq polymerase (Takara). PCR amplifications were conducted on an ABI2720 Thermocycler (Applied Biosystems, CA, USA) under the following conditions: initial denaturing at 94 °C for 5 min; 35 cycles of 30 s at 94 °C, 30 s at the annealing temperature for each designed specific primer, 30 s at 72 °C; and finally 8 min at 72 °C. The PCR products were resolved on an ABI 3730 DNA sequencer (Applied Biosystems). The N_A_ and PIC were calculated for each primer pair and population according to Botstein *et al.* [12]. In addition, the Nei’s genetic diversity (also known as expected heterozygosity) was calculated using the software GENOTYPE AND GENODIVE [13]. Furthermore, the linkage disequilibrium (LD) between loci and deviations from Hardy-Weinberg equilibrium (HWE) were tested for each locus according to Saltonstall [14] using Fisher exact tests with GENEPOP [15].

## 4. Conclusions

The eighteen microsatellite loci developed in this study provide us with an initial set of molecular markers to investigate the genetic diversity and spatial population genetic structure of *A. amurensis*. In addition, most of these markers showed transferability to closely related species, suggesting their usefulness to study polyploidy events in the genus *Anemone*.

## Figures and Tables

**Table 1:** Characteristics of 18 microsatellites for *Anemone amurensis*. Shown for each primer are the loci names, forward (F) and reverse (R) primer sequence, repeat motif (Repeat), size of cloned allele (Size), annealing temperature (Ta), Nei’s genetic diversity (N_GD_), number of alleles for each locus (N_A_), polymorphism information content (PIC) and GenBank accession numbers (GenBank). N_GD_ marked with an asterisk indicates significant deviation from Hardy–Weinberg equilibrium (*p* < 0.01).

Primer	Primer sequences (5′-3′)	Repeat	Size (bp)	Ta	N_A_	N_GD_	PIC	GenBank
BH84	F: TTGCCATGGACCAATACTCG R:GTCAGTGCAAGAAAGTAGCTGC	(TG)_9_	172	48	6	0.91	0.59	JQ518375
BH86	F: CAACCTTGCAAACCCCCTCA R: CAAAAGTCGTCGTCACCTCC	(TG)_16_	209	48	4	0.82	0.71	JQ518376
BH112	F: GCATAAGGAGTAGTCATTTCA R: CCGCAAAGGTATATATATGTG	(AC)_21_	218	52	1	0.00	0.00	JQ518377
BH206	F: TGTTGTTTCCCTTACTTGCC R: CATCTTATGTCACACTTGGG	(GT)_22_A (TG)_14_	157	48	6	0.50	0.36	JQ518378
BH235	F: CATGGCCATTGGTATCAAAC R: TTGGTGGAACAACTTAGCCC	(GT)_5_A (TG)_16_	156	48	7	0.84	0.69	JQ518379
HS27	F: GGAAGCATCATCTCACCTAC R: TTCTAGTTTTGACTGGGAGG	(AC)_7_	182	50	4	0.66	0.71	JQ518380
HS37	F: ACACAGATTCCACTCACCAC R: ACCATATTAGGCATCTCGGG	(TC)_7_ (AC)_10_	198	50	8	0.87	0.57	JQ518381
HS47	F: CACACGCAAACAGAAACACA R: GCTTGAGGTTTCATGATACAG	(TG)_22_	309	50	1	0.00	0.00	JQ518382
HS60	F: CATCATGTGCATTGGTGTCT R: GATGCTAGGAGACCAGTCTA	(GT)_18_	154	50	1	0.00	0.00	JQ518383
HS117	F: GAACACATCATTCATAGAGC R: TCCGATACAGTTTGACACTT	(GT)_6_	284	50	1	0.00	0.00	JQ518384
HS177	F: GAAAATGTGACCGTCCCTAC R: TGTCATTGGCTCACCACCTT	(AC)_7_	194	48	3	0.52	0.61	JQ518385
HS191	F: GGAGAGTGGTGTAATACCCG R: ACACTGATGTGGGCAAGGTC	(TG)_21_	272	48	1	0.00	0.00	JQ518386
HS199	F: GAGTGGAAGATCTGTGCAGG R: AGTGTGGGGTGAAACTCCTA	(CA)_8_	199	50	7	0.86 ^*^	0.70	JQ518387
HS256	F: CTGTTCCTCCGATGGCGTTT R: ACCTTACCCTTCCCCTCTTC	(TG)_7_	211	50	5	0.76	0.50	JQ518388
HS263	F: ACCAACTCACACACCAAATA R: GATCGTGATGACAAGGAGAA	(TG)_7_	299	50	1	0.00	0.00	JQ518389
HS283	F: ATGAGATGGGGATTTATGCC R: CCTTTCGGGCTTTACAACCT	(GT)_6_	183	50	1	0.00	0.00	JQ518390
HS316	F: ACTTGGGAGGTTGTTTTTGG R: CAAACTTGACTCGACACCTC	(TG)_6_	189	50	4	0.74	0.54	JQ518391
HS321	F: TGTGGAGGAAGAAGATGGTC R: GAGTGCCGCAAGATTGACAT	(CA)_8_	321	52	4	0.90	0.61	JQ518392

**Table 2:** Results of initial primer screening in *Anemone amurensis*. Parameters shown for each pair of primer are the number of the samples (N), number of alleles (N_A_), Nei’s genetic diversity (N_GD_) and polymorphism information content (PIC).

Locus	Kuandian (*N* = 15)			Langxiang (*N* = 20)			Dunhua (*N* = 17)		
	N_A_	N_GD_	PIC	N_A_	N_GD_	PIC	N_A_	N_GD_	PIC
BH84	5	0.87	0.71	6	0.84	0.67	5	0.94	0.57
BH86	4	0.41	0.65	4	0.86	0.71	4	0.70	0.68
BH112	1	0.00	0.00	1	0.00	0.00	1	0.00	0.00
BH206	6	0.52	0.83	6	0.00	0.83	6	0.54	0.84
BH235	5	0.00	0.25	6	0.93	0.64	7	0.81	0.75
HS27	4	0.85	0.73	3	0.23	0.66	4	0.67	0.70
HS37	7	0.90	0.68	5	0.87	0.46	4	0.78	0.55
HS47	1	0.00	0.00	1	0.00	0.00	1	0.00	0.00
HS60	1	0.00	0.00	1	0.00	0.00	1	0.00	0.00
HS117	1	0.00	0.00	1	0.00	0.00	1	0.00	0.00
HS177	2	0.17	0.50	3	0.66	0.54	3	0.00	0.65
HS191	1	0.00	0.00	1	0.00	0.00	1	0.00	0.00
HS199	4	0.80	0.73	5	0.91	0.76	4	0.77	0.50
HS256	3	0.95	0.45	5	0.67	0.50	4	0.81	0.53
HS263	1	0.00	0.00	1	0.00	0.00	1	0.00	0.00
HS283	1	0.00	0.00	1	0.00	0.00	1	0.00	0.00
HS316	3	0.69	0.48	3	0.79	0.66	4	0.67	0.47
HS321	3	0.67	0.49	4	0.71	0.38	4	0.88	0.66

**Table 3:** Cross-species amplification of the 18 microsatellite markers in five other species of the genus *Anemone*. For each primer pair, the number of individuals tested (N), monomorphic (M), polymorphic and number of alleles (P) and no-specific product (-) are given.

Locus	*A. raddeana* (*N* = 4)	*A. silvestris* (*N* = 4)	*A. umbrosa* (*N* = 4)	*A. reflexa* (*N* = 4)	*A. cathayensis* (*N* = 4)
BH84	P (3)	P (3)	P (2)	P (2)	P (3)
BH86	-	-	-	-	-
BH112	M	M	M	M	M
BH206	-	P (4)	P (4)	P (2)	P (5)
BH235	P (4)	P (4)	P (4)	P (5)	-
HS27	-	M	M	M	-
HS37	-	-	M	P (2)	P (2)
HS47	-	-	P (2)	M	M
HS60	-	-	M	M	-
HS117	M	M	M	M	M
HS177	-	-	P (2)	P (2)	P (2)
HS191	M	M	M	M	M
HS199	-	-	-	P (2)	M
HS256	P (5)	-	-	P (2)	P (3)
HS263	P (2)	M	P (2)	P (2)	P (2)
HS283	M	-	M	M	M
HS316	-	-	-	P (2)	-
HS321	P (2)	-	P (2)	P (2)	P (3)
